# Confirmation and fine-mapping of a major QTL for resistance to infectious pancreatic necrosis in Atlantic salmon (*Salmo salar*): population-level associations between markers and trait

**DOI:** 10.1186/1471-2164-10-368

**Published:** 2009-08-07

**Authors:** Thomas Moen, Matthew Baranski, Anna K Sonesson, Sissel Kjøglum

**Affiliations:** 1 Aqua Gen AS, Trondheim, Norway; 2 Nofima Marine, Ås, Norway; 3 CIGENE – Centre of Integrative Genetics, University of Life Sciences, Ås, Norway

## Abstract

**Background:**

Infectious pancreatic necrosis (IPN) is one of the most prevalent and economically devastating diseases in Atlantic salmon (*Salmo salar*) farming worldwide. The disease causes large mortalities at both the fry- and post-smolt stages. Family selection for increased IPN resistance is performed through the use of controlled challenge tests, where survival rates of sib-groups are recorded. However, since challenge-tested animals cannot be used as breeding candidates, within-family selection is not performed and only half of the genetic variation for IPN resistance is being exploited. DNA markers linked to quantitative trait loci (QTL) affecting IPN resistance would therefore be a powerful selection tool. The aim of this study was to identify and fine-map QTL for IPN-resistance in Atlantic salmon, for use in marker-assisted selection to increase the rate of genetic improvement for this trait.

**Results:**

A genome scan was carried out using 10 large full-sib families of challenge-tested Atlantic salmon post-smolts and microsatellite markers distributed across the genome. One major QTL for IPN-resistance was detected, explaining 29% and 83% of the phenotypic and genetic variances, respectively. This QTL mapped to the same location as a QTL recently detected in a Scottish Atlantic salmon population. The QTL was found to be segregating in 10 out of 20 mapping parents, and subsequent fine-mapping with additional markers narrowed the QTL peak to a 4 cM region on linkage group 21. Challenge-tested fry were used to show that the QTL had the same effect on fry as on post-smolt, with the confidence interval for QTL position in fry overlapping the confidence interval found in post-smolts. A total of 178 parents were tested for segregation of the QTL, identifying 72 QTL-heterozygous parents. Genotypes at QTL-heterozygous parents were used to determine linkage phases between alleles at the underlying DNA polymorphism and alleles at single markers or multi-marker haplotypes. One four-marker haplotype was found to be the best predictor of QTL alleles, and was successfully used to deduce genotypes of the underlying polymorphism in 72% of the parents of the next generation within a breeding nucleus. A highly significant population-level correlation was found between deduced alleles at the underlying polymorphism and survival of offspring groups in the fry challenge test, parents with the three deduced genotypes (*QQ*, *Qq*, *qq*) having mean offspring mortality rates of 0.13, 0.32, and 0.49, respectively. The frequency of the high-resistance allele (*Q*) in the population was estimated to be 0.30. Apart from this major QTL, one other experiment-wise significant QTL for IPN-resistance was detected, located on linkage group 4.

**Conclusion:**

The QTL confirmed in this study represents a case of a major gene explaining the bulk of genetic variation for a presumed complex trait. QTL genotypes were deduced within most parents of the 2005 generation of a major breeding company, providing a solid framework for linkage-based MAS within the whole population in subsequent generations. Since haplotype-trait associations valid at the population level were found, there is also a potential for MAS based on linkage disequilibrium (LD). However, in order to use MAS across many generations without reassessment of linkage phases between markers and the underlying polymorphism, the QTL needs to be positioned with even greater accuracy. This will require higher marker densities than are currently available.

## Background

Genomics is beginning to make an impact on animal breeding, by providing DNA markers linked to genes affecting phenotypic traits. Such markers can be used in marker-assisted selection (MAS), selection based partly or fully on DNA marker genotypes. Among the traits relevant for MAS, disease resistance traits are of particular interest, since the phenotypic measurement of these traits is often difficult or expensive and may cause animal suffering.

In aquaculture, disease resistance traits are of particular importance. In intensive culture systems, opportunities for avoidance or escape are minimal. Furthermore, interactions between fish and microbial pathogens that may be harmless under natural conditions often result in disease problems in aquaculture systems because of the added stress from biological, physical and chemical factors [1]. In contrast to farm animals, the animal strains used in aquaculture are usually very recent derivatives of wild strains [2], and therefore have had little time to adapt to the new disease pressures.

The Atlantic salmon (*Salmo salar*) is, as its relative the rainbow trout (*Oncorhyncus mykiss*), an important species in modern aquaculture. In the wild, this carnivorous species spends its first 2–5 years in fresh water, but migrates to the sea following the salt-water-acclimatising process known as smoltification. The animal spends 1 to 4 years in the ocean, and then returns to its home river to spawn, burying the eggs in the gravel substrate. The recently hatched fish, termed alevins, live of their yolk sack before they enter the fry stage and emerge from the substrate. The fresh/salt-water (anadromous) life-style of the fish is reflected in aquaculture production, where the fish spend their first 1/2 to 1 year in freshwater tanks and 2 to 3 years in sea cages until they reach market size.

Pathogens are a major problem in salmonid farming as in other areas of aquaculture. In European production of Atlantic salmon, the viral disease infectious pancreatic necrosis (IPN), caused by a double-stranded RNA virus, has for some time ranked among the diseases causing the largest losses [3,4]. In farmed Atlantic salmon, the disease causes mortality at both the freshwater (fry) stage and at the post-smolt stage shortly after transfer to seawater. The disease causes necrosis of pancreatic cells and liver cells, resulting in lethargy and sudden mortality. IPN is an endemic disease that affected 40–70% of all Norwegian seawater salmon-farming sites during the years 1994–2004, and 30–40% of freshwater hatcheries during the same period [5]. Mortality during outbreaks has been estimated to be 10–20% on average, varying from zero to almost 100% at individual sites [5]. Vaccines against IPN have been developed and are being used, but the protection is variable and not complete [6]. In the wild, the virus does not seem to cause mortalities, although wild fish may be carriers of the virus.

The Atlantic salmon has been vital to the application of modern animal breeding to aquaculture. In the early 1970s, a breeding programme was established in Norway, based on fish from different Norwegian rivers [7]. Later, other breeding programmes have been initiated in several countries [8]. Typical traits under selection are growth rate, age at sexual maturation, filet colour and other quality traits, as well as disease resistance traits. Selection for disease resistance may be based on field trials or on controlled challenge tests, but in either case, challenge-tested fish are not allowed as breeding candidates, and the breeding values of the candidates are based solely on survival rates of siblings and other relatives. Criteria for direct selection among candidates, in the form of quantitative trait loci (QTL) for resistance against various diseases, have been sought after for some time in Atlantic salmon and rainbow trout [9-24]. Of particular relevance for IPN in Atlantic salmon was a recent report on the mapping of QTL for resistance against IPN in post-smolts of Scottish origin, based on data from a field trial [25]. In that study, a major QTL was detected that explained 21% of the phenotypic variation in the data set. The QTL was found to be unusually reproducible in the population, segregating in 7 out of 20 mapping parents investigated.

Here, we report on results from a project that has been running partly in parallel with the Scottish study, reproducing the finding of one major QTL by means of a genome scan on salmon of Norwegian origin. We also present the testing of the QTL at the fry stage and the fine-mapping and further characterisation of the QTL in an extensive genetic material. The results represent, within aquaculture, a rare example of an investigation of a QTL at the level of an entire breeding population, yielding results that are directly applicable for MAS within that population.

## Results

### Phenotypic data

**Figure 1:**
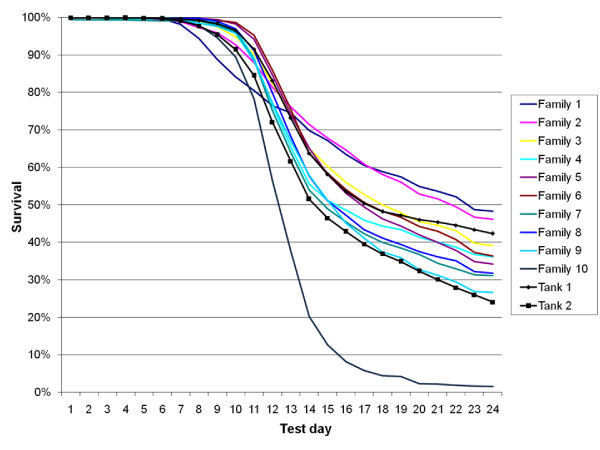
**Mortality curves from post-smolt challenge test**.

Ten large full-sib groups of post-smolt from a Norwegian breeding company (Aqua Gen Ltd., Trondheim, Norway) were IPN-challenged-tested by cohabitant challenge in two tanks. The survival curves were found to be very similar across the two tanks, and followed the expected trajectories for an IPN challenge (Figure 1). The overall mortalities in the two tanks were 70.5% and 77.8%, respectively. Mortality rates of individual families ranged from 51.7% to 98.5% (Figure 1).

### Genome scan

A genome scan was performed using the 92 first to die and 92 of the survivors (or last to die) within each full-sib family, as well as genotypes on 136 microsatellite markers spanning most of the Atlantic salmon genome (Additional file 1). The information content was relatively high in most regions (Additional file 2). Two genome-wide significant QTL for IPN resistance were identified, one having a major effect and one having a more minor effect. In addition, several suggestive QTL were identified (Table 1, Additional file 2). The major QTL, located on linkage group 21, was responsible for 75.8% of the phenotypic variation in the data set, translating into 24.6% of the phenotypic variation after selective genotyping had been taken into account, and consequently 70.3% of the genetic variation. The one other genome-wide significant QTL was located on linkage group 4, and was responsible for 0.9% of the genetic variation after selective genotyping had been taken into account.

**Table 1:** Significant and suggestive QTL for resistance to IPN

Linkage group	Position (cM)	F	No of informative parents	Chromosome-wise p-value	Proportion of phenotypic variance
21	6	43.34†	19	≈ 0†	24.6%
4	9	2.44*	20	0.001	0.9%
13	18	2.37	18	0.003	0.9%
22	65	2.31	19	0.007	0.9%
10	27	2.26	19	0.008	0.8%
17a	1	2.25	18	0.004	0.8%
25	7	2.21	19	0.003	0.8%
2	49	2.08	20	0.010	0.7%
11	44	2.04	19	0.009	0.7%
3	31	2.02	20	0.005	0.7%
17b	14	2.00	17	0.018	0.6%
12	0	1.82	20	0.049	0.5%
28	1	1.79	19	0.047	0.5%
24	0	1.78	19	0.024	0.5%
16	0	1.61	20	0.045	0.4%

*P < 0.05 (genome-wide)

†P ≈ 0 (P-value too low to be determined by permutation testing)

QTL were defined as suggestive if they were chromosome-wise significant at P < 0.05, while not experiment-wise significant at P < 0.05. The F ratio has degrees of freedom equal to the number of informative parents and the number of records (offspring) minus two times the number of informative parents [38]; the latter ranging from 3177 to 3737 depending on the number of marker-informative offspring. The proportion of phenotypic variance is the proportion of phenotypic variance explained by the QTL, after correction for selective genotyping.

### Linkage-based fine-mapping of major QTL using post-smolts

**Figure 2:**
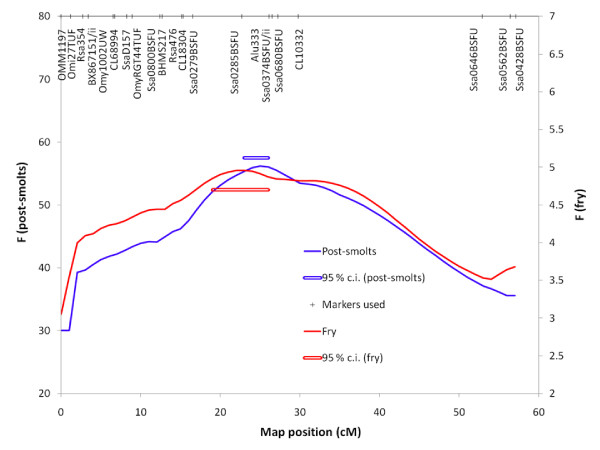
**Fine mapping of major IPN-resistance using post-smolt and fry material**. The DNA markers used are indicated at the top of the graph. The 95% bootstrap confidence intervals for position is indicated by horizontal bars.

To more precisely define the position of the major QTL on linkage group 21, 18 additional microsatellite markers were genotyped in the post-smolt individuals (Table 2). The most likely QTL position was found to be at 25 cM, with the 95% confidence interval for position being at 23–26 cM (Figure 2). The QTL now explained 29% of the phenotypic variation after correcting for selective genotyping, translating into 83% of the genetic variation. Out of the 20 mapping parents, 10 were found to be QTL heterozygous (P < 0.05). Out of the 10 QTL heterozygous parents, seven were notably more significant (P-values ranging from 5 × 10^-9 ^to 1 × 10^-36^) than the three others (P-values ranging from 0.01 to 0.04).

**Table 2:** Microsatellite markers on linkage group 21 used in this study

Marker	Position on female map (cM)	Position on male map (cM)
OMM1197	0	0
Omi27TUF^a^	1.2	> 50
Rsa354	2.7	> 50
BX867151/ii^a^	3.4	> 50
Omy1002UW	6.5	> 50
CL68994	6.7	> 50
SsaD157^a^	8.2	> 50
OmyRGT44TUF	8.9	> 50
Ssa0800BSFU	12.4	> 50
BHMS217	12.7	> 50
Rsa476	15.1	> 50
CL18304	15.3	> 50
Ssa0279BSFU	16.5	> 50
Ssa0285BSFU	22.7	> 50
Alu333	26.1	> 50
Ssa0374BSFU/ii	26.5	> 50
Ssa0680BSFU	27.2	> 50
CL10332	29.8	> 50
Ssa0646BSFU	52.9	> 50
Ssa0562BSFU	56.4	> 50
Ssa0428BSFU	57.1	> 50

^a^Markers used in the genome scan

### Test of QTL-effect in fry

Challenge-tested fry, full-sibs of the post-smolts used for the genome scan, were genotyped to investigate whether the QTL had the same effect at the fry stage as at the post-smolt stage, the disease causing mortalities at these two quite different life stages. Chi-square tests performed on groups of fry corresponded well with chi-square tests performed on their post-smolt siblings (Pearson's correlation coefficient = 0.72, P < 0.001). Of the 10 mapping parents found to be QTL heterozygous based on post-smolt data, seven were identified as QTL heterozygous in fry; these were the seven being most significant for the QTL in post-smolts. Vice versa, one parent was found to be QTL heterozygous based on fry data but not on post-smolt data, although it was close to being declared QTL heterozygous in the post-smolt test as well (P = 0.051).

### Linkage-based fine-mapping of major QTL using fry

The results had indicated that the major QTL had similar effects on fry as on post-smolts, meaning that genetic material from the fry challenge tests, in which all families in the Aqua Gen breeding nucleus had been tested, could be used to fine map and further characterise the QTL. Consequently, 108 additional full-sib groups of challenge-tested fry, representing 178 parents, were genotyped using markers located on linkage group 21. The position of the QTL based on fry data corresponded well with the position based on post-smolt data, the 95% confidence interval for position being at 19–26 cM (Figure 2). The QTL explained 16.9% of the phenotypic variation in the data set following correction for selective genotyping, translating into 48.4% of the genetic variation. Out of 178 mapping parents, 72 were found to be segregating the QTL (P < 0.05). Combined linkage disequilibrium/linkage analysis (LDLA) was performed, but could not add to the precision of the QTL position estimate (data not shown). It should be noted that fry were not scanned for QTL outside of linkage group 21. Thus, QTL located outside this linkage group, having an effect on fry but not on post-smolts, could not be detected.

### Test for population-level association between markers and the underlying polymorphism

**Figure 3:**
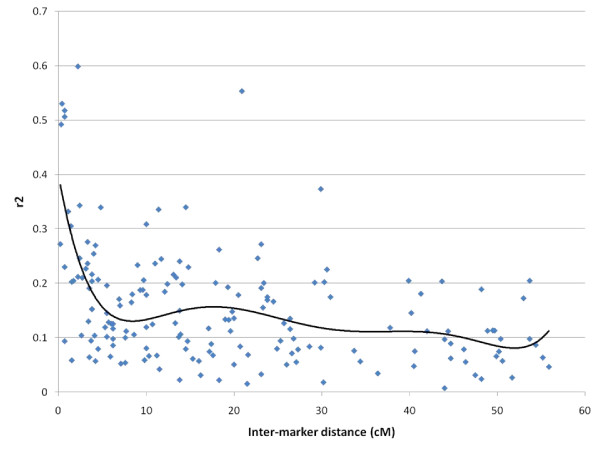
**Levels of inter-marker LD on linkage group 21 plotted by genetic distance**. The measure of LD is the square of the correlation coefficient, r^2^. All possible paired combinations of the 21 genotyped microsatellites were plotted. A 6^th ^degree polynomial was fitted to the graph.

Levels of linkage disequilibrium (LD) between markers in the QTL region showed that there was significant LD between closely linked markers on the linkage group as a whole (Figure 3). Within the QTL region, inter-marker r^2 ^values as high as 0.53 were found (Table 3). These findings indicated that there could be substantial LD between markers in the QTL region and the polymorphism underlying the QTL. Therefore, markers in the QTL region, as well as haplotypes made from these markers, were tested for their ability to predict alleles at the underlying polymorphism. This analysis was based on the 72 parents found to be QTL heterozygous, since alleles at the underlying polymorphism, and the linkage phases between alleles at the underlying polymorphism and marker alleles, were known for these parents. A four-marker haplotype was found to be the best predictor of alleles at the underlying polymorphism (Table 4); out of 41 alleles found among the 72 QTL heterozygous parents at this haplotype, only three were not exclusively linked to either *Q *(high-resistance allele) or *q *(low-resistance allele) (Additional file 3). When linkage phases deduced between *Q*/*q *and the four-marker haplotype alleles were extrapolated on the 78 remaining mapping parents with known haplotypes (28 parents had missing marker data), 11 parents were found to be *QQ*, 13 were *Qq*, and 54 were *qq*. Thus, a significant homozygote excess (Pearson's *X*^2 ^for deviation from Hardy-Weinberg expectations = 21.18, P < 0.0001) was found among the parents that were *a priori *believed to be QTL homozygous based on linkage analysis (i.e. on the earlier test for QTL based on challenge-tested offspring). Taken together, these results showed that the four-marker haplotype was a good, but not perfect, indicator of allele at the underlying polymorphism. They also gave credit to the one-gene, two-allele model that had implicitly been assumed.

**Table 3:** Levels of LD (r^2^) between markers in the vicinity of the major QTL for IPN-resistance

	Ssa0279BSFU	Ssa0285BSFU	Alu333	Ssa0374BSFU/ii	Ssa0680BSFU
Ssa0279BSFU	-	0.13	0.19	0.31	0.12
Ssa0285BSFU	6.2	-	0.13	0.15	0.08
Alu333	9.6	3.4	-	0.53	0.33
Ssa0374BSFU/ii	10	3.8	0.4	-	0.52
Ssa0680BSFU	10.7	4.5	1.1	0.7	-

Upper diagonal: r^2^; lower diagonal: inter-marker distance (cM)

**Table 4:** Test of the association between marker/haplotype alleles and alleles at the variation underlying the QTL

Marker/haplotype	No of alleles	F	P-value
Ssa0279BSFU/ii	3	2.77	0.07
Ssa0285BSFU	6	2.94	0.01
Alu333	6	7.96	1.30E-06
Ssa0374BSFU/ii	21	6.66	6.04E-12
Ssa0680BSFU	5	18.60	3.01E-12
Ssa0279BSFU-Ssa0285BSFU-Alu333-Ssa0374BSFU/ii	41	25.85	4.54E-38
Ssa0279BSFU-Ssa0285BSFU-Alu333-Ssa0374BSFU/ii-Ssa0680BSFU	47	21.99	1.28E-34

### Characterisation of the underlying polymorphism at the population level

**Figure 4:**
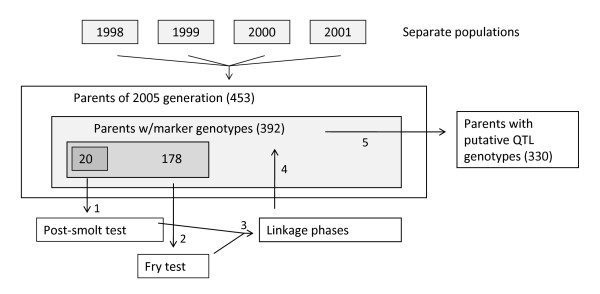
**Animals used in the study**. The animals referred to as parents in this study were parents of the 2005 generation of the breeding nucleus of an Atlantic salmon breeding company, and also parents of offspring group that were challenge-tested for IPN at the fry and post-smolt stages. These parents originated from four separate populations (or more specifically, year-classes of separate populations). Each population (i.e. year class) is denoted according to year of hatching. In the figure, the numbers refer to number of parents, and every box located within another box is a subset of the larger box. The numbers at the arrows indicate the progress of the study.

**Figure 5:**
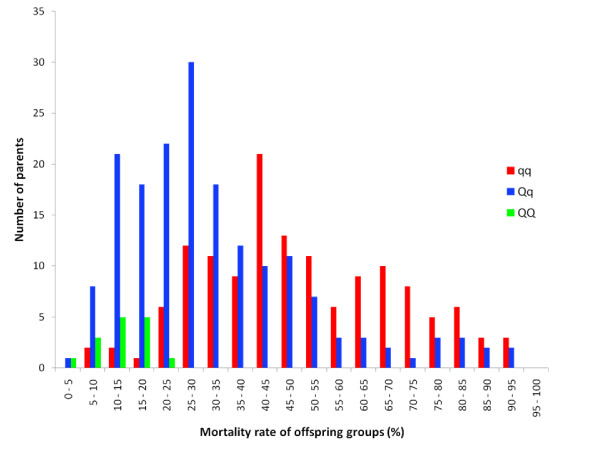
**Distribution of challenge test mortality rates among offspring of *QQ*, *Qq*, and *qq *parents**. Mortality rates of half-sib groups in the fry challenge test, classified according to the deduced genotype of the common parent at the underlying polymorphism. Each half-sib group contains two full-sib groups, and half-sib mortality rate is the average of the two full-sib mortality rates.

We now were in a position where we could characterise the segregation of the underlying gene at the population level. An additional 214 animals from among the 2005 broodstock (i.e. the parents of the 2005 generation; Figure 4) were therefore genotyped for the four-marker haplotype. Unphased haplotype data from these animals were combined with phased data from the 178 previously investigated mapping parents to deduce haplotypes at the population level, whereupon alleles at the underlying polymorphism were inferred on the basis of haplotypes found in *Qq *animals (with the three ambiguous haplotypes being classified according to the most prevalent relationship within that haplotype). For 330 out of a total of 392 genotyped broodstock animals, both alleles at the underlying polymorphism could be deduced (Figure 4). Of these, 15 were *QQ*, 177 were *Qq*, and 138 were *qq*, leading to a population level allele frequency estimate for *Q *of 0.30. Genotype at the underlying polymorphism had a highly significant (P < 10^-21^) effect on survival rates of offspring, with the average progeny mortality rates for parents with the *QQ*, *Qq*, and *qq *genotypes being 0.13, 0.32, and 0.49, respectively (Figure 5). The dominance effect was non-significant, indicating a purely additive mode of inheritance. The genotype of the underlying polymorphism was responsible for 23% of the trait variance in this test.

## Discussion

The genome scan for QTL affecting IPN resistance confirmed a major QTL explaining nearly all of the genetic variation for this trait. This implies that, in this population, the genetic component of resistance to IPN is largely under the control of one QTL, presumably corresponding to one gene, a surprising find given that disease resistance traits are generally presumed to be complex. The results agree remarkably well with the other study identifying a major QTL for IPN-resistance in Atlantic salmon [25]. In both studies the QTL explained ~25% of the phenotypic variation, it segregated in a large fraction of mapping parents (7 out of 20 parents in the Scottish study; 72 out of 176 'fry' mapping parents in the present study), and the estimated QTL positions were highly similar. Only a single marker (Alu333) was shared between the two studies in the vicinity of the QTL, but the position of this marker relative to the QTL peak was very consistent. Thus, it seems reasonable to assume that the two studies have in fact identified one and the same QTL.

In terms of the minor QTL identified, on the other hand, the overlap between the two studies was less striking. In the Scottish study, one other genome-wide significant QTL was detected, located on linkage group 26, in addition to a chromosome-wide significant QTL in linkage group 19. Of these, only the QTL on linkage group 26 was found to be harbour a (suggestive) QTL in the present work (linkage group 26 in Houston et al. [25] corresponds to linkage group 28 from the present work). The one other genome-significant QTL identified in the present study was identified on linkage group 4. These differences could be due to differences between the populations studied, with some polymorphisms segregating in one populnation but not in others, or they could be related to sampling since each population was represented by only a limited number of mapping parents in the genome scans. Also, one cannot rule out that some of the QTL could be false positives.

The major QTL on linkage group 21 was found to explain a large fraction of the phenotypic variance in both the post-smolt and fry data sets. In general, the fraction of variance explained by a QTL detected in a genome scan tends to be overestimated due to the so-called Beavis effect [26-28]. It should be noted, however, that the Beavis effect gets less severe as the experimental power increases and as the observed fraction of variance increases. Thus, our own calculations based on the formulas presented by Xu [28] showed that, for a QTL explaining 28% of the genetic variation, the bias would be almost negligible even if only 100 offspring had been genotyped. Similarly, given the sample sizes of this study, the upward bias would be insignificant even for QTL of very minor effects. We would argue that the fraction of variance explained by the major QTL on linkage group 21 is more likely to have been underestimated, since the correction for selective genotyping assumes that the 9% least and 9% most resistant post-smolts were genotyped, while in reality the set of "most resistant animals" was drawn randomly from the ~25% of animals that survived within each family. The fraction of genetic variance explained by the QTL on the post-smolt stage was calculated on the basis of the heritability estimated in fry, since a good estimate of the heritability at the post-smolt stage was not available. The heritabilities at the two life stages are not necessarily identical.

Body weight has been found to be correlated with immune functions in some studies (e.g. [29,30]), an argument for the use of body weight as a covariate in analysis for disease resistance QTL. In this study, individual fish could only be weighed during or after the challenge test, and we were thus not able to include weight as a covariate in the test for QTL (since surviving fish lived longer and thus could have put on extra weight during the challenge). We did, however, analyse the data for body weight QTL, using the 'affected/resistant' status of the fish as a fixed effect in that analysis (Moen et al., in prep.). Linkage group 21 was found not to harbour experiment-wide significant QTL for body weight, meaning that the observed QTL for IPN resistance is not a correlated effect of a QTL for body weight.

For ease of presentation, segregation data from male and female mapping parents were combined in QTL analysis, but only female map distances were used. In large parts of the Atlantic salmon genome, recombination does not occur in males [31], meaning that males do not contribute to the local positioning of QTLs in such regions. In other regions, male recombination rates are very high relative to female recombination rates. The F curves for linkage group 21 exemplify these particularities well; the sharp increase in the F statistic between OMM1197 and Omi27TUF is caused by these two markers being genetically unlinked in males, even though the female genetic distance between them is only 2 cM. For other parts of the linkage group, no male recombination events were found in the whole data set.

The major QTL for IPN-resistance on linkage group 21 had a strong effect in both post-smolts and in fry. These are very distinct life stages, given that fry live in fresh water and post-smolts in salt water. The overlapping confidence intervals for QTL position and the concurrence of QTL genotypes and linkage phases between fry and post-smolt stages strongly indicates that the same gene controls IPN resistance at both life stages, although one cannot rule out that two different, but linked genes determine resistance at the two life stages. A functioning adaptive immune system is most probably developed at a later stage than that of the experimental fish in the fry test [32], indicating that the gene underlying the QTL is likely to pertain to the innate immune system. Controlled challenge tests represent somewhat of an 'artificial' environment compared to natural farming conditions, and may lack certain natural stressors that could influence the pattern of resistance to a pathogen. The major QTL on linkage group 21, however, does not appear to be affected by such environmental effects, since it was found to have a large effect both in a field trial [25] and in an experimental trial.

According to inter-marker levels of r^2^, the extent of linkage disequilibrium increase with decreasing inter-marker distance from 5 cM. The increase in LD with decreasing distance is more pronounced in this study than in an earlier study, where the post-smolt mapping families were used to calculate levels of LD across all linkage groups [31]. This is probably due to a higher density of markers being used in the present study relative to the previous study. In spite of significant amounts of LD being present between markers in the QTL region, combined linkage disequilibrium/linkage analysis (LDLA) could not increase the QTL mapping resolution (data not shown). Possibly, the marker density in the QTL region needs to be increased before the QTL can be positioned with significantly more accuracy. At present, additional markers in the QTL region are not available.

Based on four-marker haplotypes found in QTL-heterozygous parents, putative genotypes at the underlying polymorphism were deduced in parents that had been found negative in the test for QTL. As expected, a highly significant surplus of homozygous genotypes was found, although there were 13 discrepancies in the form of parents that were negative in the test for QTL while being subsequently deduced to have the heterozygous genotype. Of these 13 parents, four carried copies of the three haplotypes alleles that were ambiguous in terms of being linked to *Q *or *q*. These four, therefore, were more likely to be true homozygotes representing ambiguous haplotypes. For the remaining nine parents, no apparent reason for their discrepancy could be found.

The QTL for IPN-resistance has now been detected in two different populations, Norwegian and Scottish, with high heterozygosity observed in both. This raises the interesting question of how an allele with a highly negative effect on a trait can be retained in populations at such large frequencies (especially since the mode of inheritance appears to be purely additive). One possible explanation is that the low-resistance allele has a positive effect on another trait under natural or artificial selection in these populations. This hypothesis, however, is opposed by an almost complete lack of observed negative genetic correlations between IPN-resistance and other traits recorded by Aqua Gen and other breeding companies. More specifically, among 14 recorded traits IPN resistance was found to be negatively and significantly correlated with only one trait. The trait in question was not among those that have been under strong selection, and the (genetic) correlation was also not strong (r = 0.17) (Aqua Gen Ltd., unpublished data). It is quite possible, therefore, that this QTL is neutral under wild conditions, but non-neutral within an aquaculture setting. Disease pressures are highly different within these two settings, and IPN-resistance has in Norway been subject to artificial selection for at most two salmon generations, with only family selection being employed and IPN being only one of several traits in the breeding goals; probably not strong enough selection for large changes in allele frequency to occur.

Given that a single QTL explains such a large proportion of the genetic variance for this trait, it is reasonable to assume that the high-resistance allele will move towards fixation relatively rapidly even without MAS, although MAS can accelerate the process significantly, or permit more focus to be put on other traits without compromising the genetic gain for IPN resistance. Some researchers might argue that loss of phenotype-affecting genetic variation should be avoided, even if the allele going towards extinction has not been found to have positive effects on other traits. In such a case, the marker tools described in this paper could be used to deliberately retain the low-resistance allele at low frequency in the population.

The QTL presented here has been in use in the Aqua Gen breeding programme since 2007, when within-family MAS was done on 30 half-sib groups in order to select the most IPN-resistant fish as parents of the elite fish (i.e. the fish used for egg production). At the time, MAS could only be done within family, and only within offspring of parents that had already been screened for segregation of the QTL. Presently, MAS could be done on the bulk of the population using linkage information, since QTL genotypes and marker-QTL linkage phases were established for 72% of the parents of the current generation (a number that can be increased by adding genotypes). There is also a potential for MAS based on linkage disequilibrium, since haplotypes were found that were associated with the underlying gene at the population level. However, the markers contributing to the haplotype cover a 10 cM interval, and are not likely to be stable over generations. Thus, there is a need to further characterise the QTL in order to obtain the most efficient tool for MAS, and to uncover the gene that is underlying this trait. Recent development in genomics, including an Atlantic salmon SNP-chip containing 16,000 SNPs developed at the Centre of Integrative Genetics (CIGENE; Ås, Norway), could prove highly useful in this respect. Also, samples of Aqua Gen broodstock dating as far back as the early 1980s will be investigated in order to test the hypothesis of the high-resistance allele having increased in frequency due to aquaculture breeding.

## Conclusion

In conclusion, the study described here led to the confirmation and fine-mapping of a major QTL for resistance against the viral disease IPN in Atlantic salmon. Alleles at haplotypes, constructed from microsatellite markers located in the QTL region, were found to be associated with resistance at the population level, providing a tool for MAS at the population level. To our knowledge, this is the first tool for LD-based tool for MAS developed in any aquaculture species. Further studies are now progressing, aiming at the identification of the underlying polymorphism.

## Methods

### Genetic material, challenge tests

The challenge tested fish were from the 2005 year class of the breeding company Aqua Gen AS. The parents of this year class came from four different, semi-closed, Aqua Gen populations originating from different Norwegian rivers in the early 1970s [7]. Two challenge tests were performed; one at the fry stage (four days past first feeding, first feeding occurring approximately four months past fertilisation and two months past hatching) and the other at the post-smolt stage (eight months past first feeding). In the fry-stage test, the standard test performed by the breeding company, all families from the year class were tested. In the post-smolt test, 10 selected full-sib families were tested. The selection of full-sib families for the post-smolt test was based upon family survival (most families selected being average, with one high-ranking and one low-ranking family also represented). Both challenge tests were performed at the VESO Vikan Research Station in (Namsos, Norway).

For the fry test, full-sib family groups of Atlantic salmon fry were transported from Kyrksæterøra (Norway) to VESO Vikan at Namsos (Norway). The fish were first-fed one day after arrival at Vikan, and acclimatised for four more days before challenge. The families were challenged in individual 10 liter tanks (84–126 individuals per tank). The challenge test was initiated four days past first feeding and terminated at 38.9% overall mortality. The fish were challenged with IPN virus (isolate V-1244) using bath challenge (3 ml of 1.35 × 10^8 ^TCID/ml added to each tank). Dead or moribund fish from 50 randomly sampled tanks were tested for IPN infection by employing an agglutination test on ascites fluid [33] or by histopathology. All diagnostic tests for IPN were positive. The fish were fed twice a day during the challenge test. From each tank, the first 10 to die and 10 random chosen survivors were sampled and stored on 96% ethanol. DNA was extracted using the DNeasy 96 kit from QIAGEN (Venlo, the Netherlands).

The post-smolt test was performed on fish that were siblings (same spawning) of the fish from the fry test. In order to induce smoltification in fish destined for the post-smolt test, the fish were exposed to 24 h daylight for the last 6 weeks prior to challenge. Sea-water-ready, PIT-tagged, Atlantic salmon smolts (8506 individuals, average weight 71 g, eight months past first feeding) were transported from Kyrksæterøra (Norway) to Namsos, (Norway) and distributed into 2 tanks (3 m diameter). Following acclimatisation for 6 days in fresh water and 3 days in salt water, 950 Atlantic salmon (average weight 58 g) infected with IPN (serotype V-1244) by intraperitoneal injection (0.5 ml per animal, 6 × 10^7 ^TCID_50 _per ml) were added to each tank. Dead fish were sampled and registered on a daily basis. The test was terminated 25 days post challenge. During challenge, the fish were fed *ad lib*. Ninety randomly sampled test fish were tested for IPN-infection using ELISA [34], and for secondary bacterial infection using bacteriological tests. All of these 90 fish were found to be IPN-positive, and negative for bacterial pathogens. Individual fish were weighed after challenge. Liver samples from challenge-tested animals were kept at -20°C. DNA was extracted using the DNeasy 96 kit from QIAGEN (Venlo, the Netherlands).

### Genome scan

A genome scan for IPN-resistance QTL was performed on the 10 post-smolt challenged families. Within each full-sib family, only the 92 first to die and 92 randomly chosen survivors, out of 1000 challenge-tested individuals, were genotyped (selective genotyping; [35]). For the genome scan, 136 microsatellite loci were genotyped (see Additional file 1 for locus names, primer sequences, accession numbers, map positions, and multiplexes). Most of these microsatellites were selected from the 2006 version of the SALMAP female linkage map (present version: [36,37]), and they were selected collectively for optimal genome coverage. In addition, a number of tetra-nucleotide microsatellites that were not on the SALMAP were included in the set. Since the published map [31] contains SNP markers that were not used in the present study, map positions of markers used in the present study do not always correspond to the positions published earlier [31]. Additional file 1 contains map positions according to the present study and to the published map [31]. Female map distances were used.

The genome scan data were checked for non-Mendelian segregation and double recombinants (indicative of genotyping errors) as laid out in Moen et al. 2008 [31]. The data were tested for QTL using the method of Haley and Knott [38], as implemented in the half-sib module of the program package QTL Express [39]. The Haley and Knott method tests parents individually for segregation of QTL alleles, and combines these independent tests into an overall test statistic. Survival was coded as a binary trait (affected/resistant) rather than as days-in-test, since the variation in days-in-test within the two binary classes was very small compared to the variation between the classes. It has been shown that a binary trait can be analysed using QTL mapping methods intended for quantitative traits, as long as the trait is a threshold trait with an underlying normal distribution [40-42]. To accommodate joint analysis of male and female mapping parents, the data set was duplicated prior to analysis, with the designation of parents as sire or dams inverted in the duplicate. Chromosome-wide significance levels were determined by within-chromosome permutation testing with 10,000 iterations [43]. A QTL was found to be genome-wide (i.e. experiment-wide) significant if the chromosome-wide significance level was smaller than 0.05 * L/1436, where L cM is the genetic length of the linkage group (i.e. chromosome) and 1436 cM is the genetic length of the whole genome. QTL that were chromosome-wide but not genome-wide significant were regarded as 'suggestive' QTL. Confidence intervals for QTL position were determined by bootstrap analysis with ≥ 1000 iterations [44]. The proportion of phenotypic variance explained by the QTL was calculated as 4*(1-MSE_full_/MSE_reduced_) where MSE_full _is the mean squared error of the full model, accommodating one QTL effect for each informative mapping parent, while MSE_reduced _is the corresponding mean squared error of the reduced model omitting QTL effects [38]. Selective genotyping was corrected for as proposed by Darvasi and Soller [35]. More specifically, the correction factor γ_p _was calculated to be 3.24 for the smolt material and 3.67 for the fry material; the uncorrected variance proportions were divided by this factor to obtain the corrected variance proportions. For the calculations, it was assumed that the 9.2% least and 9.2% most resistance animals were genotyped for post-smolts (7.7% upper and 7.7% lower for fry). Since in reality, the set of "most resistant animals" were drawn randomly from the ~25% (average) of animals that survived within family, the factor is likely to be an underestimate in the post-smolt data set. The proportion of genetic variance explained by the QTL was found by dividing the proportion of phenotypic variance with the heritability, estimated to be 0.35 based on the 2005 fry challenge test (Aqua Gen AS, unpublished data). A heritability estimate for IPN-resistance in post-smolts was not available.

### Test for QTL on individual parents

To identify QTL-heterozygotes, individual mapping parents were tested for segregation of a major QTL at individual markers or haplotypes using a two-way chi-square test. A parent was defined as QTL heterozygous if this test was significant at P < 0.05.

### Linkage-based fine mapping of QTL using post-smolt (genome scan) material and fry material

To map a QTL on linkage group 21 with more precision, 18 additional microsatellites mapping to this linkage group were identified using the SALMAP map (Roy Danzmann, pers. comm., [36]) The identities, primer sequences, multiplexes and annealing temperatures of the microsatellites can be found in Additional file 1. Microsatellite genotyping and QTL analysis were performed as for the genome scan.

### Construction of haplotypes, determination of linkage between haplotypes and QTL-alleles, and estimation of inter-marker linkage disequilibrium

Haplotypes were constructed from sets of microsatellites defining the region of a major QTL. This was done using all fry data and a custom-made computer program described in [31]. Briefly, the program performed these steps (within every mapping parent): 1) Start at the first informative marker from one end of the linkage group; 2) find the linkage phase between that marker and the next informative marker, minimising the number of recombination events in the offspring; 3) proceed in this manner to find the linkage phase between all informative marker, and thus to build the two haplotypes; and 4) for monomorphic markers, insert the same allele in both haplotypes. To link individual haplotype alleles, or single marker alleles, to alleles at the underlying polymorphism, QTL-heterozygous parents of the challenge-tested fry families (those with evidence of being QTL-heterozygous at P < 0.05) were identified, whereupon their haplotype/marker alleles were designated as being linked to the high-resistance (*Q*) or low-resistance allele (*q*) allele based on the fry data. The association between haplotype/marker alleles and the *Q*/*q *status of the chromosomes on which the alleles resided were tested for using the GLM procedure of SAS (SAS Institute Inc., Cary, NC), with allele as the only (fixed) effect in the model. The effect of deduced allele at the underlying polymorphism on survival rate of offspring groups was tested for using the GLM procedure of SAS, with fixed effects of full-sib group, number of copies of the high-resistance allele (0, 1, or 2 within every parent), and heterozygous state (0 if the parent was heterozygous for the underlying polymorphism, 1 otherwise).

The LD measure was r^2^, calculated as the square of Cramer's V [45] using the function *haploxt *of the program GOLD [46]. The sampling effect was corrected for by subtracting 1/(number of haplotypes) from r^2 ^[47]. A 6^th ^degree polynomial was fitted to the data. We did not attempt to fit a parametric function, such as the one of Sved [48], to the data, since the population is a recently admixed population [31].

## Authors' contributions

TM initiated the project, was project leader, co-supervised the laboratory work, processed raw data, did all data analysis except LDLA (AKS) and analysis of post-smolt data (contributions from SK), and wrote a draft of the manuscript with contributions from the other authors. MB co-supervised the laboratory work, developed microsatellite protocols and multiplexes, and processed raw data. AKS did LDLA. SK collected and processed phenotypic data, and coordinated the processing of genetic material. All authors read and approved of the manuscript.

## Supplementary Material

Additional file 1 **Microsatellite markers used**. Names, genome positions, PCR conditions, primer sequences, references, and accession numbers of microsatellite markers used in the study. Click here for file

Additional file 2 **Information content and statistics from genome scan**. Information content and statistics from the genome scan. Click here for file

Additional file 3 **Marker/haplotype alleles among QTL heterozygous parents classified according to genotype at the underlying polymorphism**. Marker and haplotype alleles were linked to alleles at the underlying polymorphism (*Q *= high-resistance allele, *q *= low-resistance allele) using 72 parents identified as QTL-heterozygous (P < 0.05). *Marker/haplotype alleles significantly associated with QTL alleles (binomial test, P < 0.05). Click here for file
