# Supplementary figures and images for: Confirmation and fine-mapping of a major QTL for resistance to infectious pancreatic necrosis in Atlantic salmon (Salmo salar): population-level associations between markers and trait
Source: BMC Genomics. 2009 Aug 7;10:368. doi: 10.1186/1471-2164-10-368 (PMC2728743; doi:10.1186/1471-2164-10-368)

## Slide 1
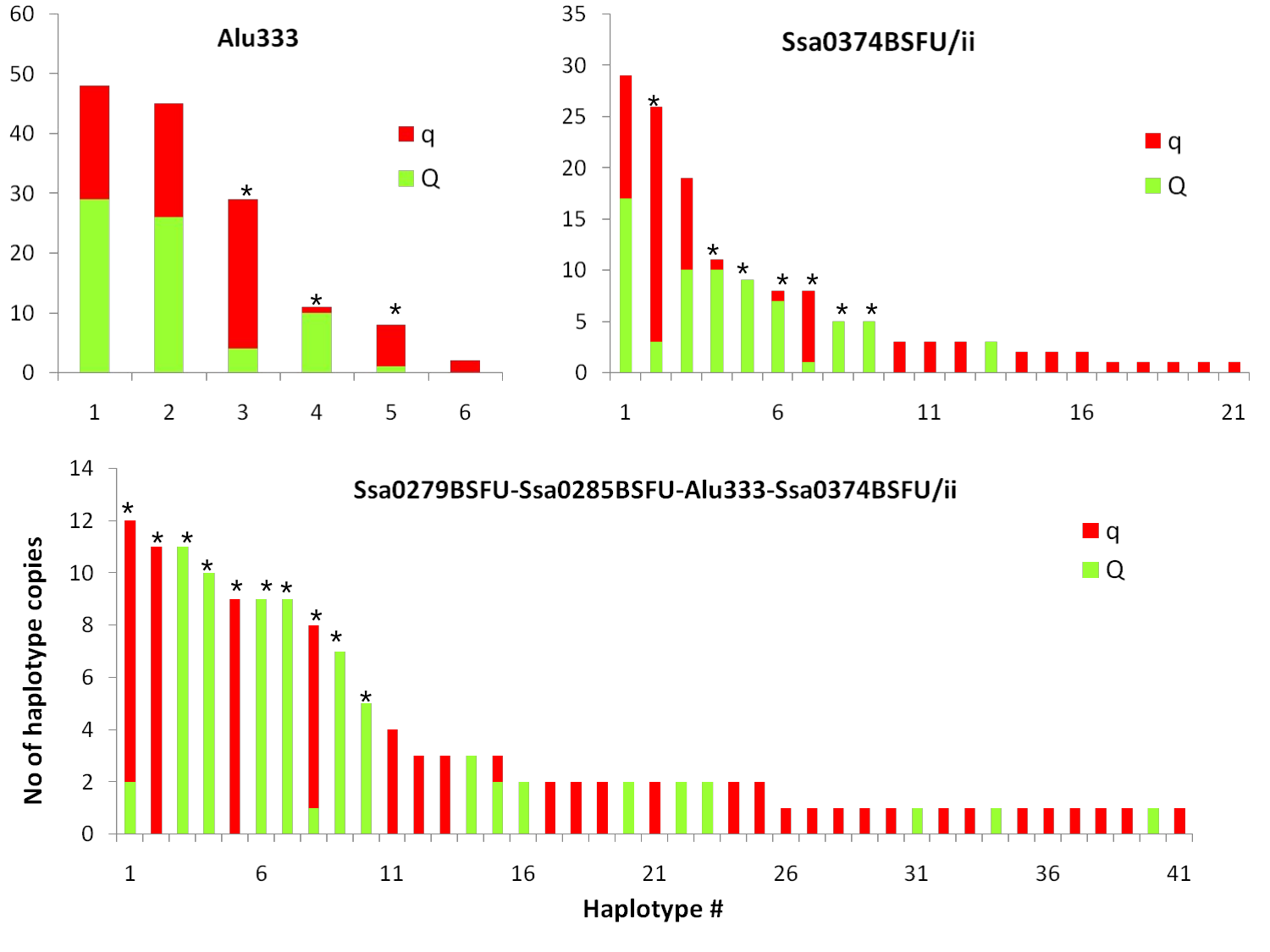

*
*
*
*
*
*
*
*
*
*
*
*
*
*
*
*
*
*
*
*

Supplement: Additional file 3 — Marker/haplotype alleles among QTL heterozygous parents classified according to genotype at the underlying polymorphism. Marker and haplotype alleles were linked to alleles at the underlying polymorphism (Q = high-resistance allele, q = low-resistance allele) using 72 parents identified as QTL-heterozygous (P < 0.05). *Marker/haplotype alleles significantly associated with QTL alleles (binomial test, P < 0.05). [file 1471-2164-10-368-S3.ppt]
